# Where have all the GPs gone – where will they go? Study of Finnish GPs

**DOI:** 10.1186/1471-2296-13-121

**Published:** 2012-12-18

**Authors:** Markku Sumanen, Tiina Aine, Hannu Halila, Teppo Heikkilä, Harri Hyppölä, Santero Kujala, Jukka Vänskä, Irma Virjo, Kari Mattila

**Affiliations:** 1 University of Tampere, Tampere, Finland; 2 Finnish Medical Association, Helsinki, Finland; 3 Kuopio University Hospital, Kuopio, Finland; 4 University of Tampere, Hospital District of Pirkanmaa, Tampere, Finland

**Keywords:** GP, Specializing, Health centre, Private practitioner, Workforce

## Abstract

**Background:**

In this paper a specialist in general practice is referred to as a general practitioner (GP). In Finland only half of all GPs work as a health centre physician. The present aim was to establish what the working places of specializing and specialized physicians in general practice are, and where they assume they will work in the future.

**Methods:**

The study population comprised 5,357 physicians licensed in Finland during the years 1977–1996. Altogether 2,956 questionnaires were returned, a response rate of 55.2%. Those either specializing (GP trainees, n=133) or already having specialized (GPs, n=426) in general practice were included in the study. Respondents were asked what kind of physician’s work they would most preferably do. They were further asked what work they assumed they would be doing in the year 2020.

**Results:**

Altogether 72% were working in public primary health centres and 14% in the private sector. Of GPs 53% and of GP trainees 70% would most preferably work in health centres. Of GPs 14% would most preferably work as private practitioners and 9% as occupational health physicians. Sixteen per cent assumed they would be working as private practitioners and 35% assumed they would be retired in the year 2020. Of GP trainees 57% assumed they would be working as health centre physicians in 2020.

**Conclusions:**

According to the present findings many experienced GPs will leave their work as a health centre physician. Moreover, several GP trainees do not consider health centre physician’s work as a long-term career option. These trends may in the future reflect a recruiting problem in many primary health centres.

## Background

General practice is the most common medical specialty in Finland. At the beginning of 2011 there were 11,621 working-age physicians with specialist licenses [1]. Of these 2,374 (20.4%) were specialists in general practice. Altogether 57% of general practice specialists were women.

In Finland physicians become licensed immediately after graduating from university. Specialist training is a postgraduate university degree including a written national final examination. Specializing requires five to six years of training. Specializing in general practice thus corresponds to that of other specialties. Qualification as a specialist in general practice requires up to two years’ work in hospitals and experience in four different specialties. The remainder of the training involves work in primary health centres. It is of note that physicians in all other specialties have to work at least nine months in primary health centres during their training. In this article GP means a specialist in general practice.

The Finnish primary health care system has some special features compared with other Nordic countries. In practice there are three different health care sectors which receive public funding: municipal health care, private health care and occupational health care [2]. GPs are qualified to work in all of these systems. The municipalities are responsible for organizing public primary health care including preventive medicine and responsibility for health of the community. However, not all physicians working in primary health centres are GPs. The only prerequisite for a permanent post in a health centre is a license as physician. In fact only one third of health centre physicians are GPs. By reason of the Finnish specializing system several hundreds of doctors in training for specialties other than general practice also work in health centres.

The fact that GPs in health centres do not receive significantly higher salaries than recently graduated licensed physicians is considered problematic. However, GPs very often have a broader job description and more administrative responsibilities than other licensed physicians. Moreover, in many health centres career possibilities for GPs are less than optimal. There are rather few senior positions, such as chief physicians, available. However, for senior positions specialization is usually needed.

The GP’s work has been assessed to be a source of considerable satisfaction [3]. Career satisfaction for GPs is associated with equity, manageable workloads and effective practice management [4]. The most frequently mentioned sources for satisfaction are a variety of work, long-lasting patient relationships, belief in the value of the work and intellectual stimulation [5]. On the other hand, longer reported working hours are associated with lower levels of satisfaction [6]. According to the expectations of future generations of physicians work conditions for GPs must be changed [7].

Health centre physicians’ work, training and salaries have improved during the last decade [8]. In Finland, however, only half of GPs work as health centre physicians [9]. Some hundred GPs also have another specialty, the most common being occupational health, geriatrics, psychiatry and internal medicine [9]. It is noteworthy that in Finland the medical care of the working-aged population may also be included in occupational health. GPs qualified in another specialty may thus have chosen their work sector according to that other. However, of GPs with general practice as their only specialty less than two in three work in primary health centres. In fact, in many Finnish health centres there is shortage of experienced physicians [10], the problem being greater in small than in large health centres [11]. Although the number of physicians in Finland is greater than ever before, the shortage in health centres has increased [12].

It is probable that the workforce problem continues in the future. Are GPs and GP trainees satisfied with their job? In order to get valuable information for health workforce planning the aim here was to ascertain what the working places of specializing and specialized physicians in general practice are, and where they assume they will work in the future.

## Methods

The Physician 2008 Study was undertaken in collaboration with the University of Eastern Finland, the University of Tampere and the Finnish Medical Association. It followed previous studies conducted in 1988, 1993, 1998 and 2003. It is thus based on previous surveys, and the same information at many time points can be compared. The survey compiled information on the social background, work history, placing on the labour market and career plans of medical professionals in Finland. It also examined physicians’ views on basic and specialist training, values and professional identity. The basic report of the Physician 2008 Study has been published by the Finnish Ministry of Social Affairs and Health [13].

**Figure 1:**
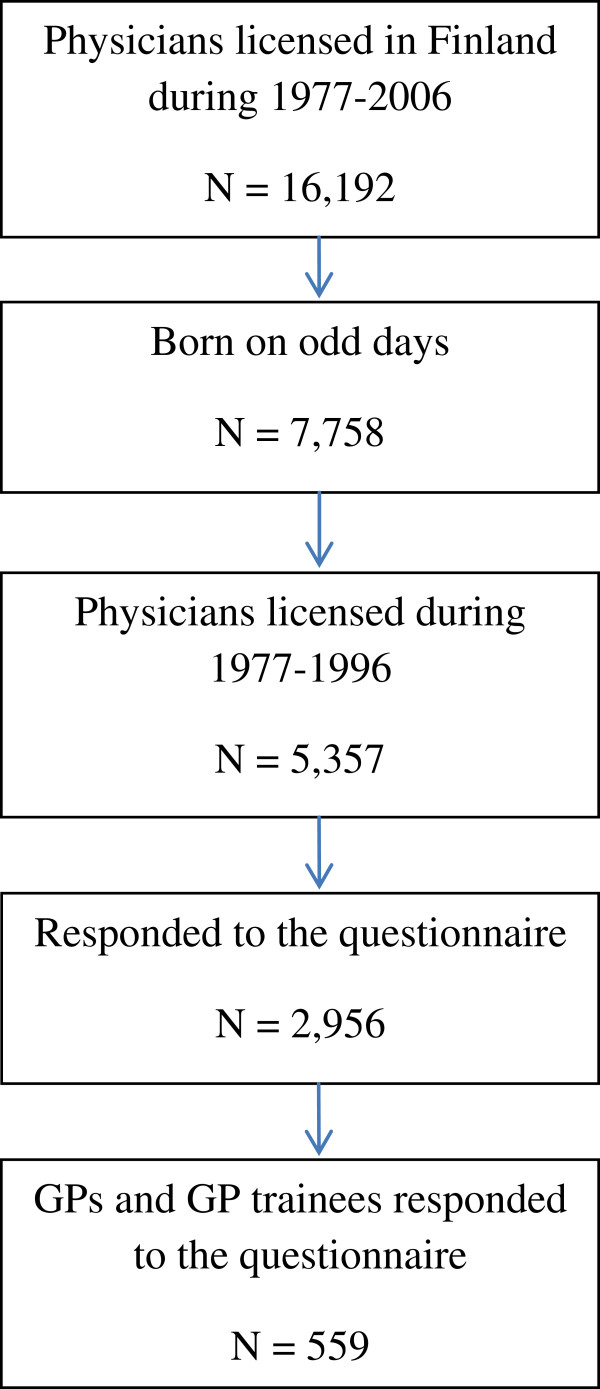
Flow chart of study population.

In the Physician 2008 Study, the basic population consisted of all physicians who were licensed in Finland during the years 1977–2006 (N=16,192). The cohort comprised 7,758 physicians, all of them born on odd days. The study population in this present analysis consisted of 5,357 physicians licensed during the years 1977–1996. The reason for this is that most of them had already completed their specialization. An electronic questionnaire was used in the first stage of data collection. A traditional postal questionnaire was sent to those whose e-mail addresses were not available and those who did not respond to the electronic questionnaire. Both postal and e-mail addresses were collected from the database of the Finnish Medical Association, which covers all physicians licensed in Finland. Altogether 2,956 responded to the questionnaire, a response rate of 55.2%. Women responded to some degree more actively than men [14]. The 559 responders are a subset of the 2,956 respondents. (Figure 1). 

Respondents were asked about their specializing situation. Those either specializing (GP trainees, n=133) or already specialized (GPs, n=426) in general practice were included in the study.

Respondents were asked whether their spouse and parents were also physicians or had another health care occupation. This was relevant since the career development is influenced by the spouse’s occupation [15]. Moreover, parents’ attitudes may also be transmitted [16,17]. Respondents were asked whether they had administration or leadership education. They were also asked whether they were working in a senior position such as chief physicians and whether they had a permanent post or position.

Job satisfaction was inquired using a 5-point Likert scale. Alternatives in respect of satisfaction were “very satisfied”, “quite satisfied”, “difficult to say”, “rather unsatisfied” and “very unsatisfied”. The respondents’ working place was recorded, the alternatives being hospital, primary health centre, private sector, university, state, and other.

Participants were asked what kind of physician’s work they would most preferably do. The alternatives were health centre physician, hospital physician, occupational health physician, private practitioner, research work, education, administrative work, out-sourced physician (employed by companies hiring physicians), or no particular preference. It was also asked what work they assumed they would be doing in the year 2020. The alternatives were the same as the above, including also retired.

Analyses were made by SPSS 16.0. The data were analysed using cross-tabulation and *χ*^2^ –test. Logistic regression analysis was used to examine factors underlying the assumption of work as a health centre physician in 2020. The factors analysed were the characteristics of physicians presented in Table 1. Those who assumed they would already be retired were not included. 

**Table 1:** Characteristics of GP trainees and GPs

	**GPs trainees**	**GPs**	**Total**
	**N = 122 − 133**	**N = 386 – 426**	**N = 498 – 559**
	**%**	**%**	**%**
Gender			
females	79	68	71
males	21	32	29
Occupation of spouse			
physician	15	23	21
other health care occupation	9	12	11
Occupation of mother			
physician	3	2	2
other health care occupation	26	16	18
Occupation of father			
physician	13	4	7
other health care occupation	5	4	4
Administration or leadership education	8	13	12
Working in a senior position	3	32	23
Permanent post or position	59	76	72

Every respondent has not answered all questions.

Research Ethics Committee of the Kuopio University Hospital has confirmed that according to the Finnish legislation, no ethical assessment or approval was mandatory for our study. The respondents agreed to participate in the study by answering the questionnaire.

## Results

The data analysed covered 559 physicians, 396 women and 163 men (Table 1). The occupation of the spouse was physician in one in four cases among GPs and one in six among GP trainees. Either parent also being a physician was somewhat rare except for GP trainees, of whom one in seven reported that their father was a physician. GPs more often than GP trainees reported having completed administration or leadership education. One third of GPs were working in a senior position, whereas among GP trainees this was rare.

### Satisfaction with work

One fifth of GPs were very satisfied with their work (Table 2). Of GP trainees the proportion was one ninth, the difference being statistically significant (p=0.021). However, the corresponding proportions of at least quite satisfied were 82% and 80%, the difference being not significant (p=0.517). 

**Table 2:** Satisfaction with job of GP trainees and GPs

	**GP trainees**	**GPs**	**Total**
	**N = 119**	**N = 421**	**N = 540**
	**%**	**%**	**%**
Very satisfied	11	20	18
Quite satisfied	69	62	64
Difficult to say	11	6	7
Rather unsatisfied	7	8	8
Very unsatisfied	2	4	3

### Present work

Most physicians specializing in general practice were working in public primary health centres (Table 3). Around two in three GPs were working in primary health centres or other municipal working places. One in six GPs were in the private sector. Some GPs also worked in a state or university position and also in some other capacity. 

**Table 3:** Proportions (%) of working places of GPs and GP trainees

	**GPs**	**GP trainees**	**Difference**	
	**N = 419**	**N = 118**		
	**%**	**%**	**% units**	**p-value**
Private sector	17	4	13	<0.001
Other position	5	2	3	0.084
State	2	0	2	0.108
University	2	1	1	0.621
Hospital	4	11	−7	0.006
Public primary health centre	70	82	−12	0.007

### Most preferable work

Of GP trainees 70% would most preferably work as a health centre physician (Table 4). Among GPs the proportion was 53%. Fourteen per cent of GPs would most preferably work as private practitioners. The corresponding proportion of GP trainees was 4%. Occupational health physician was the preference of 9% of GPs and 5% of GP trainees. Administrative work was the most attractive occupation among 8% of GPs. 

**Table 4:** “What kind of physician’s work you would most preferably do?” Proportions (%) of replies of GPs and GP trainees

	**GPs**	**GP trainees**	**Difference**	
	**N = 425**	**N = 131**		
	**%**	**%**	**% units**	**p-value**
Private practitioner	14	4	10	0.001
Administrative work	8	3	5	0.042
Occupational health physician	9	5	4	0.141
Research work	3	1	2	0.098
No particular preference	4	4	0	0.978
Hospital physician	7	8	−1	0.479
Education	2	3	−1	0.312
Out-sourced physician	0	2	−2	0.002
Health centre physician	53	70	−17	0.001

### Assuming to work in the year 2020

Of GP trainees 57% assumed they would be working as health centre physicians in the year 2020 (Table 5). Among GPs the corresponding proportion was 28%. One in six GPs and one in seven GP trainees anticipated working as private practitioners. One third of GPs assumed they would be retired in 2020. 

**Table 5:** “What job do you assume you will be doing in the year 2020?” Proportions (%) of replies of GPs and GP trainees

	**GPs**	**GP trainees**	**Difference**	
	**N = 421**	**N = 131**		
	**%**	**%**	**% units**	**p-value**
Retired	35	4	31	<0.001
Administrative work	11	5	6	0.078
Private practitioner	16	13	3	0.379
Education	1	1	0	0.932
Out-sourced physician	0	1	−1	0.073
No particular preference	1	2	−1	0.932
Occupational health physician	4	6	−2	0.258
Research work	1	3	−2	0.037
Hospital physician	3	8	−5	0.006
Health centre physician	28	57	−29	<0.001

In logistic regression analysis the only statistically significant factors for the assumption of working as a health centre physician in 2020 were female gender (OR 1.61, 95% CI 1.02 – 2.56) and permanent post or position (OR 2.78, 95% CI 1.77 – 4.38). Administration or leadership education (OR 0.32, 95% CI 0.17 – 0.61) and working in a senior position (OR 0.45, 95% CI 0.26 – 0.80) had a negative association with this assumption.

## Discussion

According to the findings only half of general practitioners would most preferably work as health centre physicians. In the year 2020 the assumed posting as GPs in a health centre was even less probable. Although one third of GPs assume they have retired many GPs intended to work as private practitioners and some in occupational health care which in Finland also includes medical care. It is of note that many private practitioners in fact work in this kind of occupational health care.

In the Physician 2008 Study, the study population included 83% of Finnish working-age physicians. Male physicians had a somewhat lower responding rate than females. The gender imbalance might have had an impact on our findings because the proportion of GPs among females is higher than among males. Another bias may be the finding that seventy percent of our GP sample work in health centres, which is higher than previous studies which report 50%. Probably some GPs qualified in another specialty work according to that other. However, we think that these matters do not substantially affect the findings in the present study and the results presented here can be generalized to the Finnish medical profession.

Moreover, although the response rate, 55.2%, could have been better, it may be still considered a good level compared to international studies [18]. Unfortunately, we do not know what proportion of the total Finnish GP population was included in this study. Therefore the response rate among GPs and GP trainees is unknown. However, according to statistics from the previous Finnish Physician surveys [19], we can estimate that the response rate of the GP trainees is as high as the overall response rate in the present survey whereas GPs’ response rate is somewhat lower.

According to one previous study, job satisfaction reduces physicians’ inclination to switch sector [20]. Obviously, the heavy workload might be one reason. Indeed, severe work strain and overload is common among GPs [21]. During recent years, the incidence of overwork and burn out has increased among GPs [22]. It is known that job stress factors are predictive of high levels of job dissatisfaction and lack of mental wellbeing [23]. A low level of job satisfaction in general practice is also a reflection of GPs feeling that the ability to control their work is low [24]. Excessive paperwork, health reforms and bureaucratic interference, excessive hours and on-call work have emerged as the major causes of stress and lack of job satisfaction among general practitioners [25]. Moreover, high job stress is associated with insufficient practice management [26]. Although low job satisfaction has been associated with a sector switch in previous studies, this was not consistent with our results.

In our study fourteen percentages of GPs would most preferably work as a private practitioner. Roughly the same number of GPs who would preferably work as a private practitioner see themselves in this role in the future.

It has been stated that physicians who regard themselves as entrepreneurs prefer to work in the private sector [27]. The choice of work sector also depends on such factors as career possibilities or leisure-time facilities [28]. Another possible explanation for switch of sector is that GPs have proved to be less committed to their organizations than other physicians, the main factors being work-related psychosocial elements such as high job demands, low job control and poor inter-colleague consultation [29]. Moreover, many health centre physicians feel that in primary health care there is a leadership problem, this being associated with a shortage of doctors, bureaucracy, work distribution and educational aspects [30]. Another reason might be equal salaries with less work and less responsibility in the private sector compared with municipal workplaces. It is also possible that some GPs are dissatisfied with opportunities to obtain sufficient continuous medical education. According to one previous study health centre physicians in Finland had less continuous medical education than other physicians [31]. It is thus understandable that some GPs prefer a more independent placing in the private sector, the importance of which as a working sector for physicians has grown over the past few years [32].

Administrative work would not appear to be popular. Only one in ten participants here assumed they would be doing that work in the future. This kind of job may be regarded as frustrating on account of bureaucracy and the incessant struggle for resources. On the other hand, higher salaries and managerial positions are often worth reaching for. It is probable that many GPs with administration and leadership education will also work as chief physicians in health centres. The finding that one third of GPs assumed they would be retired in 2020 seems logical because age has an impact on this result. It is also probable that the same age group holds the senior positions.

Neither was hospital work popular. This finding is understandable since general practice is a speciality concentrating on primary health care. One in nine GP trainees were currently working in hospitals the most likely reason being the requirement of experience in four other specialties.

In our study the intention to pursue research work was somewhat rare. This finding may be regarded as expected, since in the GP’s position there is little if any time to concentrate on it. Moreover, in general practice there is not the same tradition of research work as in other specialties. Already a decade ago it was stated that most general practitioners have no contact with research or academic general practice, few achieve higher degrees compared with hospital consultants, and there are few academic posts in general practice [33].

Our finding that many GPs plan to work elsewhere than in health centres may also be considered positive. Specializing in general practice offers a comprehensive education opening up a variety of opportunities, and this may explain why general practice is the most popular specialty in Finland. Diversity of work has also been shown to be the main factor affecting choices of specialty [34].

It may be speculated whether creating more possibilities for positive career advancement could increase willingness to remain in primary health centres. There is debate on the possibility of emphasising the role of specializing in general practice. In present-day Finland it is not necessary to be a GP in order to secure a permanent post in health centre. Perhaps the prerequisite of having specialized might increase the attractiveness of this position.

It may also be speculated whether reorganizing the public health care system in line with that of the other Nordic countries is worth aspiring to. In Sweden each county council and in Norway each municipality has to decide how to serve its population with primary care, which in both countries is mainly publicly provided [35,36]. In Denmark practicing health professionals are self-employed and reimbursed by the regions, mainly using a fee-for-service system [37].

This kind of survey concentrating on assumption of working places in the future has not been done in Finland before. Our findings are thus new and may also be considered important for workforce planning. Follow-up with qualitative work is needed to explore the potential reasons further.

## Conclusions

According to the present findings many experienced GPs will leave their work as a health centre physician. One third will be retiring and some prefer a more independent work as a private practitioner. Another concern is that several GP trainees do not consider health centre physician’s work as a long-term career option. These trends may thus in the future reflect a recruiting problem in many primary health centres. Further research is required to determine underlying factors.

## Competing interests

The authors declare that they have no competing interests.

## Authors’ contributions

MS carried out the statistical analysis and drafted the manuscript. TA helped to draft the manuscript. HHa, TH, HHy, SK, JV, IV and KM conceived the study, participated in its design and coordination and critically revised the article. All authors have read and approved the final manuscript.

## Pre-publication history

The pre-publication history for this paper can be accessed here:

http://www.biomedcentral.com/1471-2296/13/121/prepub
